# On-Device Mobile Visual Location Recognition by Using Panoramic Images and Compressed Sensing Based Visual Descriptors

**DOI:** 10.1371/journal.pone.0098806

**Published:** 2014-06-03

**Authors:** Tao Guan, Yin Fan, Liya Duan, Junqing Yu

**Affiliations:** School of Computer Science and Technology, Huazhong University of Science & Technology, Wuhan, People's Republic of China; Xiamen University, China

## Abstract

Mobile Visual Location Recognition (MVLR) has attracted a lot of researchers' attention in the past few years. Existing MVLR applications commonly use Query-by-Example (QBE) based image retrieval principle to fulfill the location recognition task. However, the QBE framework is not reliable enough due to the variations in the capture conditions and viewpoint changes between the query image and the database images. To solve the above problem, we make following contributions to the design of a panorama based on-device MVLR system. Firstly, we design a heading (from digital compass) aware BOF (Bag-of-features) model to generate the descriptors of panoramic images. Our approach fully considers the characteristics of the panoramic images and can facilitate the panorama based on-device MVLR to a large degree. Secondly, to search high dimensional visual descriptors directly on mobile devices, we propose an effective bilinear compressed sensing based encoding method. While being fast and accurate enough for on-device implementation, our algorithm can also reduce the memory usage of projection matrix significantly. Thirdly, we also release a panoramas database as well as a set of test panoramic quires which can be used as a new benchmark to facilitate further research in the area. Experimental results prove the effectiveness of the proposed methods for on-device MVLR applications.

## Introduction

Mobile visual location recognition (MVLR) [1]–[6], [37]–[46] is a kind of location-based service that can be used in a variety of contexts, such as hygiene, outdoor object search, entertainment, work, personal life, etc. The function of MVLR is to enhance the user's experience by providing an augmented city guide or navigational tools. In MVLR applications, the tourist captures a query image of the landmark which he would like to know more about, the system performs recognition by using the query image and feedbacks of the observed landmark to the user for browsing purpose. It's worth noting that MVLR needs to fully consider the characteristics of mobile devices, including (i) limited computing power and battery capacity, and (ii) mobile user's fast response-time requirement [4]. Many researchers use client-server (C/S) mode to perform MVLR. High performance servers endure the computational load and speed up the recognition process. However, the transmission delay of the network may affect the user's experience [7].

Although the research on MVLR has witnessed many achievements in the past few years, the technology is still not mature enough. For example, most existing methods are based on Query-by-Example (QBE) principle which generally requires an example image as a query to search for similar database images. However, the level of retrieval reliability is still insufficient due to the likely variations in the capture conditions (e.g. light, blur, scale, and occlusion) and the viewpoint changes between the query image and the images in the database.

We think that the use of panoramas can improve the usability of on-device mobile visual location recognition systems obviously due to following reasons. Firstly, compared with a query image with relatively narrow angular field of view, a panoramic image commonly contains more useful visual information which can be used to generate visual descriptors with higher discrimination power. Secondly, the use of panoramic query image can help users to formulate their visual intent more conveniently, which is useful for enhancing the user experience.

While promising, it is not a simple task to realize panoramas based MVLR directly on resource limited mobile devices. For example, it is difficult to fit the panoramas searching engine into the RAM of mobile devices because the memory budget of an on-device MVLR application is only about dozens of megabytes. Secondly, the limited computational power makes the fast and accurate panoramic image searching become a difficult and challenging task on mobile devices.

In view of the above problems, we make following contributions in this research to the design of a panoramas based on-device MVLR system. Firstly, we use heading information from digital compass to facilitate the BOF descriptors generation process. Our approach fully considers the characteristics of the panoramic images and can facilitate the panorama based on-device MVLR to a large degree. Secondly, we propose a compressed sensing based encoding method. Our method is fast and compact enough for power limited mobile devices, which makes the storing and searching of panoramic image database directly on mobile devices come true. Thirdly, we also release a panoramas database as well as a set of test panoramic quires which can be used as a new benchmark to facilitate further research in the area.

The rest of this paper is organized as follows: Section 2 discusses the related work. Section 3 gives an overview of our panorama based on-device MVLR system. Section 4 gives the details of panoramas database construction. Section 5 introduces the heading-aware BOF method that will be used to generate the descriptors of panoramic images. Section 6 discusses the compressed sensing based visual descriptor code method. Section 7 gives the location recognition method. Section 8 shows some experimental results. Section 9 is a conclusion.

## Related Work

In the past decade, MVLR [1]–[6], [37]–[46] has attracted a lot of researchers' attention. The most popular MVLR approaches are based on client-server mode in which the image database is searched on a remote server by using the unloaded query images. A very important problem to be solved in client-server based MVLR system is to reduce the transmission delay as much as possible to ensure good user experience. In case of large size query image, some related works have been proposed to extract the image features (such as SIFT [10] and SURF [11]) on the mobile device and generate the compact image descriptor directly for transmission. For example, Ji et al. [8], [9] design an efficient location aware visual descriptor encoding scheme to compress visual descriptors for extremely low bit rate mobile visual search.

In that case, traditional image describing (such as BOF [12]) and indexing (such as inverted index) techniques commonly used in the field of CBIR (Content-based image retrieval) can be directly taken to fulfill the location recognition task. This framework has been proved to be simple and efficient in dealing with the problem of large-scale image retrieval. However, the accuracy is affected by discarding spatial information. Some related works are proposed to address this problem. Lazebnik et al. [13] design an extension of the orderless BOF by partitioning the image into increasingly fine sub-regions and computing histograms of local features found inside each sub-region. Cao et al. [14] design the Spatial-Bag-of-Features by projecting the image features to different directions or points to generate a series of ordered BOF, then selecting the most representative features to generate a new BOF-like vector representation of an image. Another approach is using phrases or collocations generated from visual words. Zhang et al. [15] use statistical methods to select visual phrases that frequently co-occurring visual word pairs. Zhang et al. [16] propose the geometry-preserving visual phrases (GVP) that models local and long-range spatial interactions between the visual words. Zhou et al. propose the spatial coding [34] to encode the spatial relationships among local features in an image, and contextual visual vocabulary [35] that takes both the spatial and semantic contexts into consideration.

Some approaches use additional information acquired from mobile devices such as GPS and various sensors. Chen et al. [4] propose a discriminative vocabulary learning method for landmark recognition based on the context information such as location from the GPS and direction from the digital compass. Guan et al. [7] integrate the information from inertial sensors into the Vector of Locally Aggregated Descriptors (VLAD [17]) generation and image similarity evaluation processes. David et al. [18] propose to perform localization by registering a single omnidirectional ground image to a 2D urban terrain model and introduce a novel image descriptor that encodes the position and orientation of a camera relative to buildings in the environment.

More recently, it is reported that large scale image search can be performed directly on a mobile device to obtain fast on-device MVLR. Guan et al. [7] develop several methods to make the city scale on-device visual location recognition come true such as: compressing image descriptors to get memory efficient searching engine, utilizing gravity for more distinctive image descriptor, and integrating GPS into the image similarity evaluation process for accurate location recognition.

For on-device visual location recognition, the storage and computation requirements for high-dimensional visual descriptors are extreme. The quantization based methods can encode an image descriptor into only several bytes while searching accuracy is acceptable. Jegou et al. [19] introduce product quantization (PQ) to compress the vector to a short code composed of its subspace quantization indices. Chen et al. [20] propose residual vector quantization based approaches that database vectors are quantized by residual vector quantizer. However, the codebook needed for these methods may be too large to fit the mobile phones, and fail to make significant improvement as code length increases. Ji et al. [36] propose a task-dependent codebook compression framework. The hashing based methods convert very high-dimensional real vectors to long binary strings. Most hashing methods can be classified into two categories: the random projection based methods and the learning based methods. The random projection based methods like Locality Sensitive Hashing (LSH) [21] have to generate long codewords to preserve the locality of the data points, leading to large storage space and high computational cost. The learning based methods like Spectral Hashing [22] work well for short codewords but fail to make significant improvement as code length increases [23]. While the vectors can be encoded to be very short, larger code sizes are needed to get the highest absolute accuracy [24].

In this paper, we explore the possibility of large binary coding using compressive sensing. The theory of compressive sensing (CS) [25]–[27] enables the recovery of sparse or compressive signals from a small set of incoherent projections. Duarte et al. [28]–[30] propose that many applications can be more efficient and accurate to extract information directly from a signal's compressive measurements than first recover the signal and then extract the information. Haupt et al. [31] show that the signal's compressive measurements can be effectively used in signal classification problems, where the goal is to identify a signal from a class of candidates. Lin et al. [24] design Compressed Hashing to process the compressive measurements without recovering. The weakness of the CS based methods is that the random projection matrix costs too much storage space when the dimension of input vector is very high.

While promising, existing methods are not efficient enough because the retrieval reliability of the Query-by-Example based image retrieval principle is still insufficient. In view of that, we propose a panorama based framework in this research to improve the performance of on-device MVLR systems.

## Architectures

**Figure 1:**
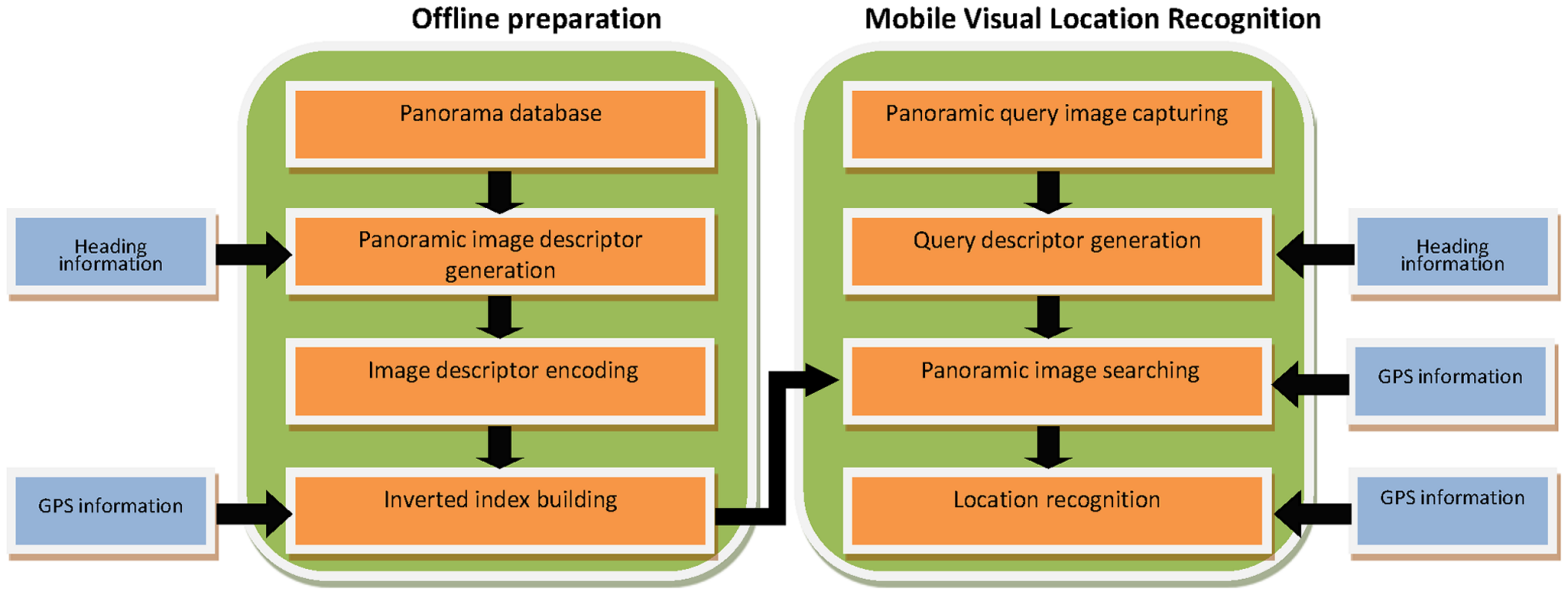
Panorama based on-device MVLR framework.

The proposed panorama based on-device MVLR framework is shown in Figure 1.

Offline Panoramic Images compression and indexing: In this stage, we build a panoramic image search engine which will be used for on-device MVLR. We firstly generate the heading-aware BOF descriptor of each database panoramic image and then convert them into binary vectors by using the method given in section 6.

Online location recognition: To perform location recognition, the heading-aware BOF descriptor of the query panoramic image will be generated firstly. Then, the method of section 7 will be used to perform panoramic image searching directly on mobile device to fulfill the location recognition task.

Subsequent sections describe the method used in detail, present the results, and evaluate the method's performance.

## Database Construction

We collect the HUST Panorama database by using a mobile mapping vehicle composed of Ladybug3 360°spherical digital video camera system, Global Positioning System (GPS), and Inertial Measurement Unit (IMU). We directly use the panoramic images to generate the database.

We collect the street view of the main roads of the Huazhong University of Science and Technology (HUST) and obtain 5081 images captured at 4-meter intervals on average. Our work is permitted by the Huazhong University of Science and Technology. The range (GPS) of our study is between (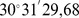, 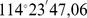), (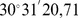, 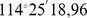), (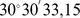, 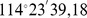) and (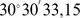, 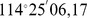). The GPS information of each panorama is recorded. The route can be simulated according to the GPS information. And the heading information of each panorama can be estimated from the route.

**Figure 2:**
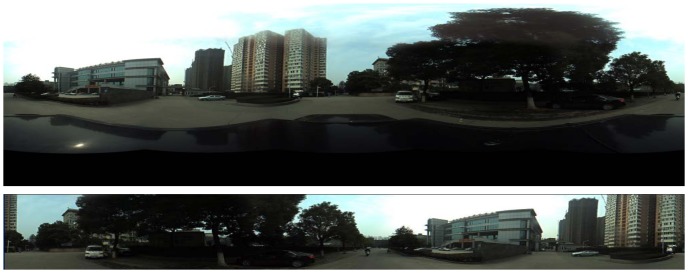
Example of the panorama in database. The panorama above is the original image obtained by Ladybug3 360°sphericaldigitalvideo camera, and the panorama upper is the image used in database.

We perform a two-step pre-treatment for database panoramas. Firstly, we segment the panoramas, removing the irrelevant part. Secondly, we rearrange each panorama according to the heading information. After rearranging, the angle between the leftmost position of panorama and the north is 0. See Figure 2.

**Figure 3:**
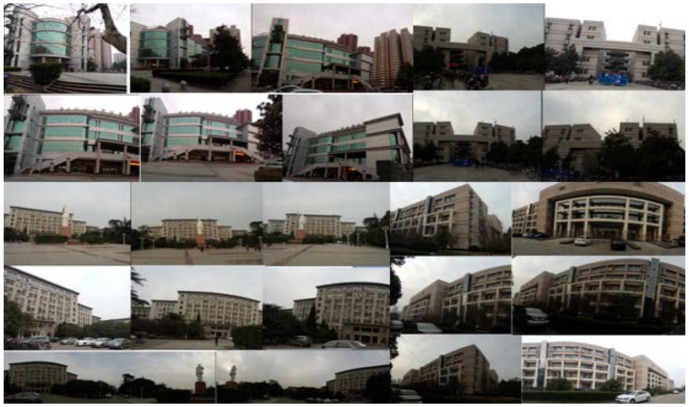
Query panoramas captured at different views.

Query panoramas are captured by camera phone after the database is built. We select 22 landmarks at the campus and capture the panoramas of each landmark at different views, including 206 queries altogether. Each query panorama is labeled manually, and the GPS and heading information is also recorded. It is worth noting that the queries may not be complete 360-degree panoramas. See Figure 3.

## Visual Descriptor Generation

This section deals with the problem of visual descriptor generation on mobile device. In this section, we will first describe the heading-aware method for visual descriptor generation, which can be applied to the panorama based on-device MVLR system, followed by the approach of generating BOF (Bag-of-features) using heading-aware method.

So many kinds of methods such as BOF (Bag-of-features), VLAD (Vector of Locally Aggregated Descriptors) and REVV (Residual Enhanced Visual Vector) can be used to generate the visual descriptors of panoramic images. Obviously, we can directly generate the descriptors of query and database panoramic images to fulfill the searching task. However, it will cause trouble in our case because the database images are complete 360-degree panoramas, while the submitted query may not be. As shown in Figure 3. If traditional method is adopted to generate the corresponding visual descriptors, the performance of location recognition will be affected because the database images contain much irrelevant information (such as another building, the street and trees). We design the heading-aware method to address this challenge.

### Heading-Aware Method for Database

**Figure 4:**
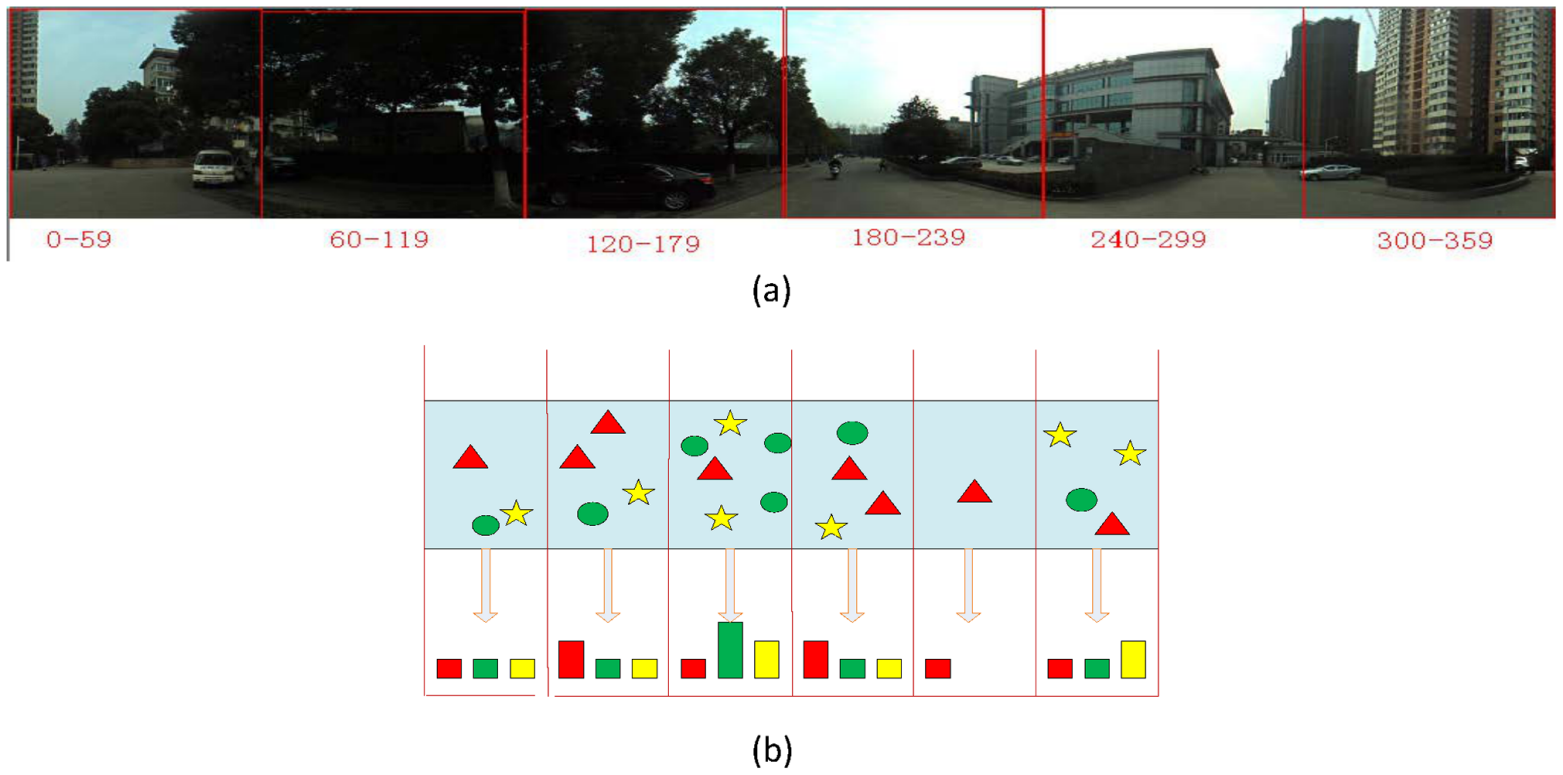
Illustration of heading-aware method for database. (a) The database panorama is partitioned into 6 parts equally. “0–59” represents that the part 1 ranges from 0 to 59 degree. (b) Illustration of heading-aware method for database. Stars, triangles, and circles represent different visual words.

The ideal situation is that we can extract the query corresponding areas only, filtering out the disturbed areas. Based on this idea, we propose to partition the database panoramas equally. As shown in Figure 4, the panorama is partitioned into 6 parts. The first part ranges from 0 to 59 degree, and the last part ranges from 300 to 359 degree. Generate the sub-descriptor for each part separately, and concatenate them to compose the heading-aware visual descriptor.

Suppose that the database panoramas are divided into L parts. The heading-aware visual descriptor is concatenated by L sub-descriptors:
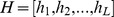



Where 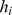 is the sub-descriptor in the i-th part.

### Heading-Aware Method for Query

**Figure 5:**
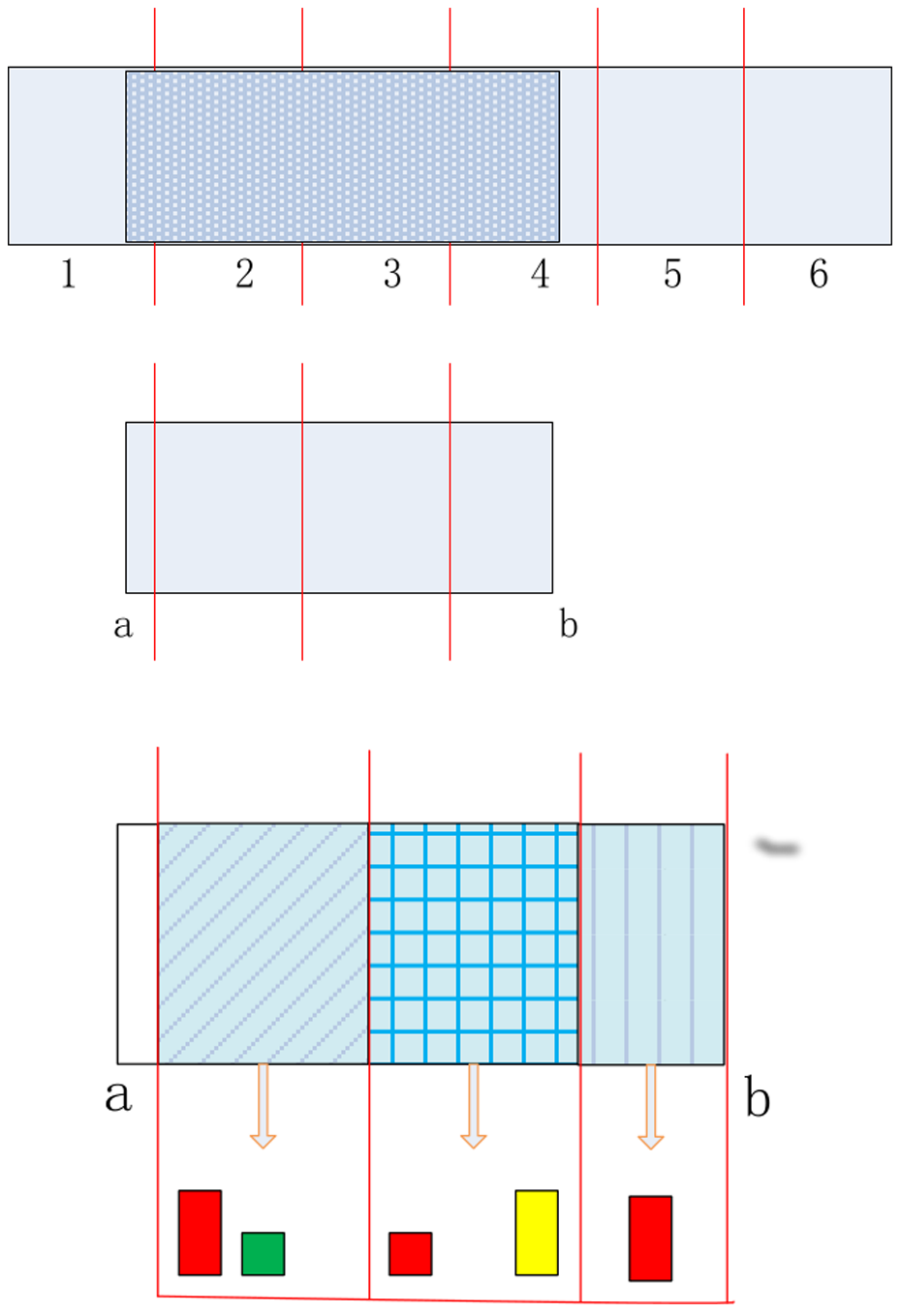
Illustration of heading-aware method for query.

While capturing the query panorama, two headings from compass are recorded. One heading is recorded when it starts to capture query panoramas, and another heading is recorded when it finishes capturing. While combining the perspective of camera, the query's start and end angles with north direction can be estimated. Suppose that the start angle with north direction is a, and the end angle is b. The query's range is defined as the interval:
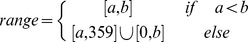



The range corresponds to the parts:
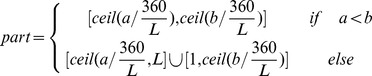



Check the area's endpoints. If the query in this part is less than half of it, the endpoint part is excluded. As shown in Figure 5, the query's range corresponds to the parts 1 to 4. Check the endpoint part 1 and part 4, and the part 1 is excluded. For each included part of query, extract the local image features and generate sub-descriptor. Suppose that the included parts are 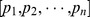. The heading-aware descriptor of query is concatenated by n sub-descriptors:
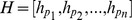



Where 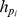 is the sub-descriptor in the i-th included part.

### Image Matching Using Heading-Aware Method

Given a specific query with included parts 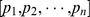, for each database panorama, only the sub-descriptors in the included parts are selected and concatenated to compose the heading-aware BOF.

Let 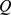 be the query, and 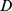 be the database panorama. Their similarity under this feature is defined as:
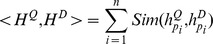



Where 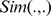 could be any histogram similarity measure, e.g. cosine similarity.

Applying the heading-aware method to panorama based MVLR system can filter out most disturbed areas. Meanwhile, partial spatial information can be retained by dividing the panoramas, which makes up for the shortcoming of traditional visual descriptor methods to some extent. However, it also leads to an increase in the descriptor dimension. More memory space is needed to store the database's descriptors, and more computation time is needed to retrieve. We suppose to address this problem by the compressed sensing based code method, which will be introduced in details in section 6.

### Heading-Aware BOF

In this research, we use the BOF method which has been proved to be the most common and effective approach in dealing with the problem of large-scale image retrieval. In BOF method, local image features from sample images are used to train a vocabulary tree [32] to generate a set of visual words for local feature quantization use. For each image, all of the detected local features are quantized to its nearest visual word by using the built vocabulary tree, and the normalized visual words histogram with components weighted by term frequency-inverse document frequency (TF-IDF) [33] will be used as the BOF descriptor for image retrieving use.

Algorithm 1 Heading-aware BOF for database

Input:


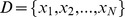
: the database;


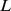
: the number of parts;


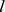
: the number of the vocabulary tree's layers;


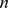
: the number of nodes at each layer;

1. Extract local image features from each panorama of 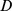;
2. Use the local features to train a vocabulary tree, and the number of leaf nodes is 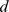, 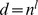;
3. Determine the part that the feature belongs to by the feature location. Generate the BOF 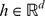 for each part of every panorama in 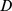, and the BOF for panorama is 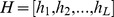, 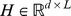.

Output:

The vocabulary tree;


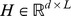
: heading-aware BOF for database 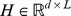.

## Bilinear Compressed Sensing Based Visual Descriptor Code

The heading-aware BOF increase the space and computation time requirements. This section deals with this problem by descriptors coding. In this section, we will first introduce the compressed sensing theory and bilinear projection briefly, followed by the approach of using bilinear method to realize the measurement of compressed sensing.

### Compressed Sensing

Compressive sensing (CS) has aroused great attention in the signal processing community. Recent theoretical results show that if the signal is sparse (or nearly sparse) in some basis, then with high probability, the observations essentially encode the salient information in the signal. We say that a signal 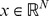 is K-sparse if it can be represented as 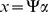 where the vector 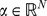 has only 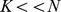 significant coefficients [29].

For a K-sparse signal, the observation is obtained by projecting the signal onto a randomly chosen vector, namely random projection, of which the entries are independent and identically distributed (i.i.d.) (Gaussian or Binary random variables, or random Fourier basis vectors). Consider an 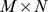 measurement matrix 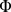, 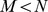. We measure 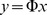 and note that 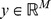 with 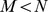. The signal 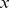 can be accurately recovered from 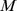 observations by solving:
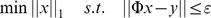



It's inspiring that CS enables the recovery of sparse or compressive signals from a small set of incoherent projections. And recent researches show that the signal's compressive measurements can be more effectively used than first recover the signal, which enable data compression. However, for high-dimension data, the random projection matrix may require too much storage space and computation time. For example, if encoding a 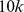-dimensional vector into a 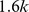-dimensional vector, the 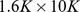 random projection matrix takes roughly 61MB, which is extreme large for on-device MVLR system. To address this challenge, we adopt bilinear projection [24] for CS.

### Bilinear Projection [24]

Let 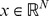 denote our descriptor vector. The projection framework for 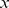 is that
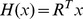
where 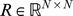 is the projection matrix.

We reorganize it into a 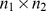 matrix with 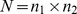:
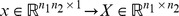



The bilinear projection uses two matrices 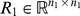 and 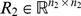:
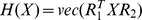



Where 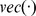 denotes column-wise concatenation.

This projection is given by 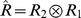, where 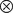 denotes the Kronecker product: 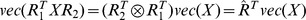
follows from the properties of the Kronecker product. Thus, a bilinear projection can be considered as a special case of a full projection, such that the full projection matrix 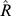 can be reconstructed from two smaller matrices 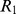 and 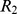, and memory and computation time are reduced significantly.

### Bilinear Compressed Sensing Code

We present a method for visual descriptor coding by using random bilinear projection. As described earlier, we adopt BOF to generate visual descriptor. Considering that the BOF vector is sparse, and the sparsity increases with dimension, we can directly extract the measurements by random matrix. Given a 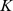-sparse BOF 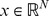, we want to convert it to a binary code 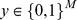 with 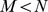.

Algorithm 2 Bilinear Compressed Sensing Code

Input:


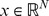
: the visual descriptor;


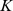
: the sparsity of 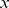;


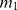
, 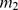: the dimension of measurement matrix;

1. Reorganize 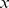 into a 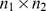 matrix 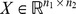 with 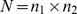.
2. Generate two linear projections 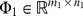 and 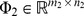 with 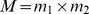, and they are independently sampled from 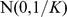 independently.
3. Compute the embedding of data 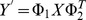, 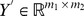.
4. Compute the binary code 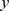 by thresholding 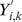 with respect to the median of each column.
5. Concatenate 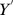 to form 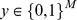.

Output:


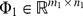
, 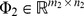: the measurement matrices;


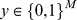
: the binary code.

The comparison of the traditional method and bilinear method is shown in the Table.1. The bilinear method can significantly reduce the space and computation time requirements. For example, converting a 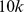-dimensional vector into a 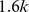-dimensional binary code, 61 MB of memory is needed for a full random measurement matrix. However, it takes only 31 KB of memory when using the bilinear measurement matrices 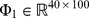 and 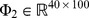.

**Table 1:** The space and time complexity contrast between full projection based CS and bilinear based CS.

	full	bilinear
Space Complexity	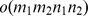	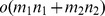
Time Complexity	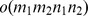	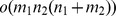

### Distance Computation for Binary Codes

Given a query, we need to compute the distance to each binary code in the database. The Hamming distance is very effective for binary codes. Taking into account the retrieval accuracy, we adopt the asymmetric distance, in which the database descriptors are binarized after compression, but the query is just compressed. For a query, which is compressed into 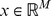, the distance to database binary code 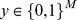 can be calculated as:
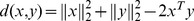



Since 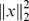 is same for all database codes, we only need to compute 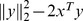, and 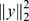 can be stored previously.

## Location Recognition Methods

This section introduces the method used to perform location recognition. Before the location recognition process, we generate the heading-aware BOF for database panoramas by Algorithm 1, and encode them by Algorithm 2. The codes
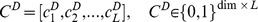
of database are stored. 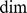 is the dimension of code. 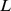 is the number of diving parts.

The heading information and GPS are used to generate the heading-aware BOF. In the similarity evaluation process, the GPS information is used to narrow down the retrieval range to improve the location recognition accuracy. The location recognition method is described in detail as follows:

Step 1) Narrow down the candidate range set 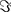 by using the GPS information of the query panorama.

Step 2) Find the included parts 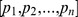 by using the heading information of the query panorama.

Step 3) Generate the heading-aware BOF 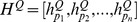, and encode it by bilinear compressed sensing. The code of query is
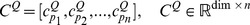



The code of query isn't binarized after the bilinear compressed sensing for improving the accuracy.

Step 4) For each database panorama in set 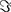, select the sub-code 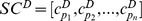. The distance between the query and each candidate in set 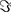 can be calculated as:
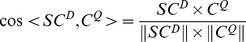



## Results and Discussion

In this section, we first give the result of our location recognition. Then we evaluate the heading-aware BOF algorithm on the HUST panorama database that we release. And we evaluate the performance of bilinear compressed sensing on the HUST panorama database and San Francisco PFI database [5] previously released. San Francisco PFI database contains 638K database images with precise ground truth labels and geotags. 803 query images taken with different camera phones are provided to test retrieval performance.

The performance of different methods are measured by recall@ R which is defined as the proportion of query vectors for which the correct match is ranked within the top R returned results.

### Location Recognition Results

We extract SURF features from each database panorama. The hierarchical K-means are adopted to train a vocabulary tree which contains 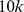 visual words for the use of heading-aware BOF generation. We divide the panoramas into 12 parts. Each visual descriptor is encoded into 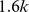 bits by algorithm 2.

To perform the location recognition, we first use the GPS information to find the database candidates within 200 meters from the query. We generate the heading-aware BOF according to the query's heading information and use the algorithm proposed in the section 7 to perform the location recognition. Finally, a geometric verification (RANSAC with a 2D affine model) [5] process is carried out to refine the location recognition results.

**Figure 6:**
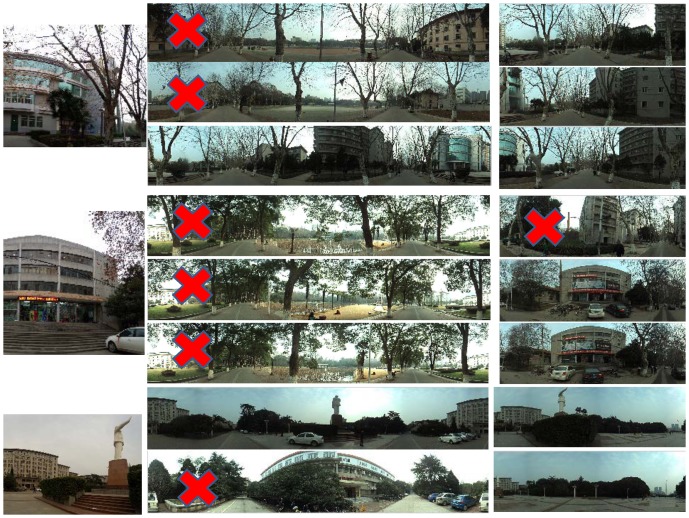
Location recognition results. (a), (b), (c), (d) and (e) are achieved by the method of [5]. (f), (g), (h), (i) and (j) are achieved by our proposed method. The image marked with red “X” denotes wrong result.

We also implement the method proposed by Chen et al. [5] for comparison. Figure 6 gives the comparison results. We can see that our method outperforms the method of [5]. The method of [5] fails in many query examples, while our method works well.

In our current system, each database panorama takes 2410 bytes (2 bytes for panorama ID, 8 bytes for GPS information, and 2400 (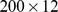) bytes for visual descriptor code). The projection matrices used in bilinear Compressed Sensing code take 30.5 KB. Thus, it takes about 12 MB to perform the location recognition.

We test the computation time spent in our location recognition method on the SONY mobile phone with a 2-core 1 GHz CPU. Table 2 shows the average computation time of each phase. Note that the query is a panorama that has a wider perspective than the ordinary image, it takes more time to detect the feature points and generate the local descriptors.

**Table 2:** Average computation time.

Step	Time(s)
Feature points detecting	3.10
Local descriptors generation	0.52
Heading-aware BOF generation and bilinear CS code	1.37
Location recognition	0.21
total	5.20

### Heading-Aware BOF Result

**Figure 7:**
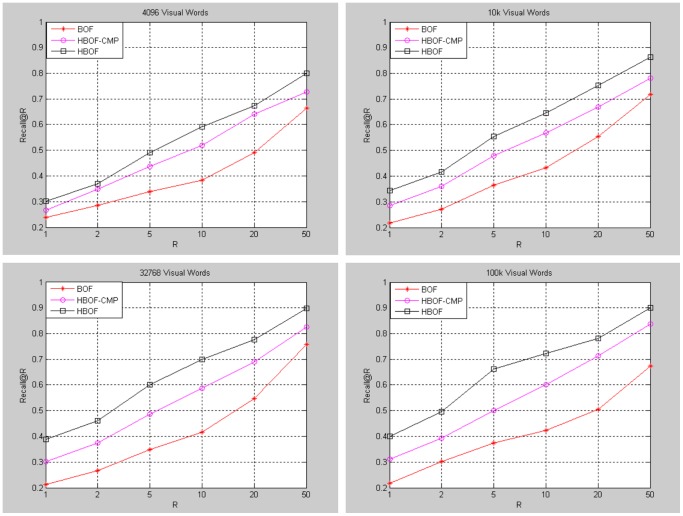
Performance of heading-aware BOF under different vocabulary size.

Our method generates database and query descriptors separately by part. To verify the effectiveness of our method (HBOF), we propose a method for comparison (HBOF-CMP). The method generates database descriptors separately by part, while query does not. Meanwhile, generate traditional 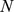-dimensional BOF for query. For database, select the descriptors of the corresponding parts and add them to a 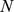-dimensional BOF. Figure 7 shows the comparison of the methods under different vocabulary size. The panoramas are diving into 12 parts. The baseline method is traditional BOF method. The performance of BOF is very poor because of lots of disturbed areas. The HBOF and HBOF-CMP methods improve the performance greatly by filtering out most of disturbed areas. Moreover, HBOF outperforms HBOF-CMP because HBOF can retain partial spatial information of database and query panorama.

**Figure 8:**
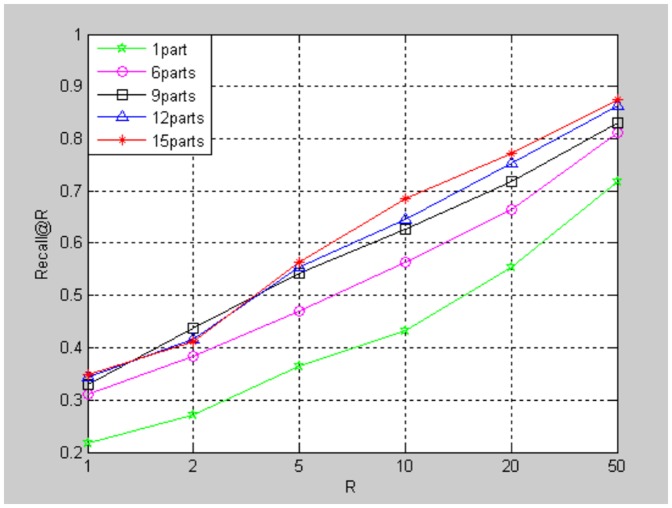
Performance of heading-aware BOF under different 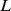.

Figure 8 shows the performance of our method under different numbers of divided parts 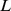. Our method turns out to be traditional BOF method when the number is 1. The vocabulary trees used contains 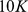 visual words. The performance upgrades when 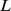
increases.

### Bilinear Compressed Sensing Results

We test the performance of bilinear Compressed Sensing based visual descriptor code on the HUST panorama database and San Francisco PFI database. For HUST panorama database, we divide the panoramas into 12 parts and generate Heading-aware BOF by algorithm 1. The vocabulary tree used contains 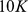 visual words, and the descriptor for each part is a 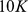 vector. So the size of each panorama is 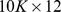. For San Francisco PFI database, we generate the BOF descriptors by the method of []. The descriptor for each image is a 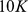 vector.

**Figure 9:**
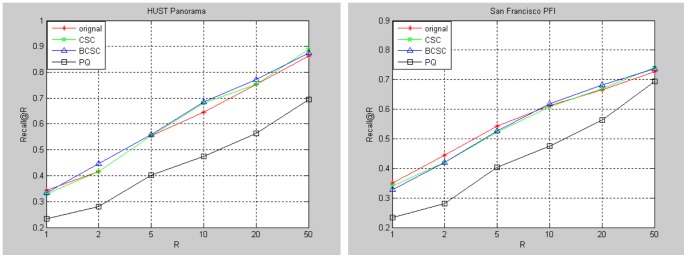
Comparison of different code methods on HUST Panorama and San Francisco PFI database.

We compare our method (BCSC) with Compressed Sensing based visual descriptor code (CSC) and Product Quantization (PQ, 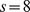 and 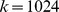, 10bytes). For BCSC and CSC, each 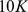-dimensional descriptor is encoded to a 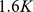-dimensional code. The projection matrix size for CSC is 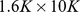, and both projection matrices for BCSC are 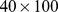 with the descriptor is reorganized into a 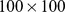 matrix. Figure 9 shows the comparison of these methods on HUST Panorama and San Francisco PFI database. The baseline (original) is the location recognition result by using the original descriptors without encoding.

Comparing our method with Compressed Sensing based method, our bilinear projection based method can reduce memory needed for projection matrix and computation time greatly. Table 3 gives the memory and time requirements comparison. When the descriptor dimension scales up to 1 M, which is a common size in many applications, the memory needed to store the projection matrix may be too large to endure. Compared to the bilinear projection matrices, only hundreds of KB is needed.

**Table 3:** The memory and time requirements comparison when encoding a 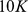 vector into a 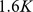 vector.

	BCSC ( 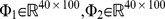 )	CSC ( 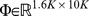 )
Memory	30.5 KB	61 MB
Time (ms)	11	30

The computation time is tested on a machine with a 4-core 2.5 GHz CPU.

Comparing our method with PQ, PQ performs poorly in our experiments. PQ can encode the descriptor into very short code, but it fails to provide a rational accuracy, which proves the necessity of larger code size for high dimensional descriptor to get the highest absolute accuracy. Meanwhile, the memory needed to store the codebooks is 39.1 MB, which is extreme for our application.

## Conclusions

In this paper, we discuss the MVLR by panoramas images. The traditional visual descriptors perform poorly on the panoramas images. We propose a heading-aware visual descriptor method for panorama image. Experimental results on HUST Panorama database prove the effectiveness of heading-aware BOF. We also design bilinear Compressed Sensing based code which reduces the costs of memory and computation time for projection matrix significantly. Experimental results on HUST Panorama and San Francisco PFI database show that the performance of bilinear projection method is comparable with Compressed Sensing based code with a full projection matrix. We also release a HUST Panorama database. We hope that the released dataset will facilitate further research in the area.
